# Digging into Soil: Effects of Soil Texture on RT-QuIC Performance for Environmental Prion Surveillance

**DOI:** 10.1021/acs.est.6c03979

**Published:** 2026-07-16

**Authors:** Stephanie J. Katircioglu, Heather N. Inzalaco, Allen Herbst, Daniel J. Storm, Stuart S. Lichtenberg, Rodrigo Morales, Reece McGinn, Daniel P. Walsh, Wendy C. Turner

**Affiliations:** † Wisconsin Cooperative Wildlife Research Unit, Department of Forest and Wildlife Ecology, 184787 University of Wisconsin-Madison, Madison, Wisconsin 53706, United States; ‡ U.S. Geological Survey, National Wildlife Health Research Center, Madison, Wisconsin 53711, United States; § Wisconsin Department of Natural Resources, Eau Claire, Wisconsin 54701, United States; ∥ Department of Veterinary and Biomedical Sciences, 70195 University of Minnesota, St. Paul, Minnesota 55108, United States; ⊥ Minnesota Center for Prion Research and Outreach, University of Minnesota, St. Paul, Minnesota 55108, United States; # Department of Neurology, 12340 The University of Texas Health Science Center at Houston, Houston, Texas 77030, United States; ∇ Centro Integrativo de Biologia y Quimica Aplicada (CIBQA), Universidad Bernardo O’Higgins, Santiago 8370993, Chile; ○ U.S. Geological Survey, Montana Cooperative Wildlife Research Unit, Wildlife Biology Program, University of Montana, Missoula, Montana 59812, United States; ◆ U.S. Geological Survey, Wisconsin Cooperative Wildlife Research Unit, Department of Forest and Wildlife Ecology, University of Wisconsin-Madison, Madison, Wisconsin 53706, United States

**Keywords:** disease surveillance, chronic wasting disease, pathogen detection, prions, prion amplification assay, soil-borne pathogens, sensitivity, specificity

## Abstract

Chronic wasting disease (CWD) is a fatal neurodegenerative disease caused by infectious prions affecting wild and captive cervids. Transmission occurs directly between hosts or indirectly through contact with prion-contaminated environments. Soils, particularly those rich in clay, are hypothesized to enhance prion stability, retention, and bioavailability. Accurate detection of prions is therefore important for understanding environmental transmission risks. Real-time quaking-induced conversion (RT-QuIC) is a sensitive assay used to detect PrP^CWD^ in tissue, excreta, and environmental materials. However, RT-QuIC performance across soil textures has not been evaluated. This study assessed RT-QuIC sensitivity and specificity using laboratory-prepared soils spiked with CWD-positive brain homogenate or water controls under a standardized extraction method. Conditional on the extraction method used, results suggest that RT-QuIC performance depends on soil texture, and thus, an optimal time-to-threshold (TTT) cutoff required to balance sensitivity and specificity will also vary with soil texture. RT-QuIC exhibited higher sensitivity and moderate specificity in soils with low clay (<20%) and moderate to high silt content, whereas high-clay soils (>20%) with low to moderate silt content (2%–60%) reduced both sensitivity and specificity, and required shorter TTT cutoffs. These findings highlight the importance of accounting for soil texture in environmental CWD surveillance.

## Graphical abstract

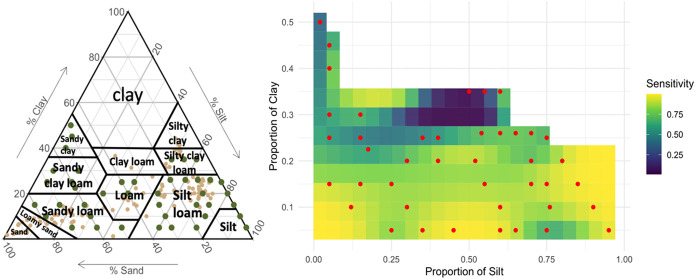

## Introduction

Soil is a highly diverse and complex natural resource composed of organic and inorganic material that supports a wide range of ecological processes.^1^ It also serves as a reservoir for pathogens such as protozoa, fungi, bacteria, viruses, and prions and may therefore play a significant role in environmental disease transmission. Examples of environmentally transmitted diseases include anthrax (*Bacillus anthracis*), hantavirus, histoplasmosis, and prion diseases such as scrapie and chronic wasting disease (CWD).^2^ Among these agents, prions^3,4^ and *B. anthracis* ^5,6^ have demonstrated years-long stability in the environment, raising concerns of persistent environmental transmission and its implications for disease management. While most nucleic acid-containing pathogens can be detected using culture and molecular-based techniques such as polymerase chain reaction (PCR), prions require alternative methods for detection due to their proteinaceous structure. Current prion detection assays are often constrained by sensitivity and specificity limitations when working with complex environmental matrixes.^7–11^ This study examines these limitations by evaluating prion detection across different soil textures to improve prion surveillance and our understanding of environmental CWD dynamics.

CWD is caused by misfolded prion proteins (PrP^CWD^) that affect captive and free-ranging individuals in the Cervidae family.^12^ Transmission occurs through direct contact between infected and susceptible individuals^13,14^ or indirectly through PrP^CWD^-contaminated materials.^15^ Environmental contamination with PrP^CWD^ is thought to result from infected individuals shedding infectious prions through urine,^7,16^ saliva,^7,16,17^ blood,^17^ feces,^18,19^ antler velvet,^20^ fetal and gestational tissue and fluids,^21–24^ semen and male reproductive tissues,^25^ as well as from carcasses,^15,26,27^ creating potential environmental foci for PrP^CWD^ accumulation and host exposure risk. Although the sources and relative importance of indirect exposure for CWD are unclear, prion-contaminated soils are hypothesized to serve as an infectious reservoir, as cervids routinely consume tens to hundreds of grams of soil per day.^28^

Prions bind to various soil components that can enhance their retention, stability, and bioavailability across the landscape, increasing the risk of indirect transmission. Prions exhibit a high affinity for silicate clay minerals^29–36^ and organic matter (OM), particularly humic acids.^33,37,38^ The binding of prions to clay minerals also has the potential to enhance prion transmissibility. For instance, when prions are bound to montmorillonite (MTE), a type of smectite clay mineral, they are significantly more infectious than unbound prions in intracerebrally or orally challenged Syrian hamsters.^29,30^ In an oral challenge study with Syrian hamsters, prion-bound MTE increased the effective infectious titer of prions by a factor of 680 compared to unbound prions.^30^ Similarly, prions bound to whole soils exhibited greater infectivity than unbound prions at equal or lower doses in an intracerebral challenge using an elk PrP transgenic mouse model.^36^ Together, these studies suggest that soil may play an important role in the environmental persistence and transmission of CWD prions.^29,39,40^

Despite the potential role of soil composition in CWD dynamics, there are few tools available to detect PrP^CWD^-contamination in soil. Current diagnostic methods include antibody-based approaches such as enzyme-linked immunosorbent assay (ELISA), as well as protein amplification assays such as protein misfolding cyclic amplification (PMCA) and real-time quaking-induced conversion (RT-QuIC). Although ELISA is regarded as the “gold standard” for *post-mortem* tissue diagnostic testing, it lacks the sensitivity necessary for unconventional sample types such as soil.^41,42^ PMCA is often considered more sensitive than RT-QuIC for detecting extremely low concentrations of prions, particularly in complex or minimally processed biological samples.^43^ Its ability to amplify infectious prions makes PMCA valuable for studying prion infectivity and transmission dynamics,^44^ especially in experimental settings where bioassays are required.^45^ In addition, PMCA protocols can be more flexible with respect to reaction conditions and substrates, which may improve detection for specific prion strains or sample types.^46,47^

While PMCA offers several advantages, RT-QuIC has emerged as more practical for routine surveillance because it provides real-time, fluorescent-based readouts, has shorter assay times, and generates noninfectious end products.^48^ Additional advantages of RT-QuIC over PMCA include its use of recombinant, non-animal-derived substrate and its high-throughput detection capabilities, an important advantage for surveillance programs working with large sample quantities.^49,50^

Over the past decade, RT-QuIC has proven reliable in detecting low levels of PrP^CWD^ in a diversity of sample types, including tissue,^51–57^ excreta,^8,10,19,58,59^ secreta,^7,60,61^ ticks,^62^ plants,^63^ environmentally relevant surfaces,^64^ and soil.^9,11,65^ While RT-QuIC is gaining broader application, its performance can be affected by inhibitors such as biochemicals,^8,42^ highly concentrated tissue homogenates,^42^ recombinant substrate properties,^49,66^ sample preparation methods,^7^ and instrument settings (i.e., temperature).^8,67^ Although significant progress has been made in improving the assay performance for biological samples, no studies have evaluated the sensitivity and specificity of RT-QuIC for detecting PrP^CWD^ across different soil textures. Understanding the limitations of prion detection in soil is essential for applying this assay to environmental PrP^CWD^ surveillance. In this study, we experimentally examined how soil texture influences RT-QuIC performance by creating synthetic mixtures of clay, silt, and sand to emulate soil textural classes found in a CWD-endemic area of southwest Wisconsin, U.S.A. These were then subjected to two separate experiments. In the first experiment, we spiked soils with a known PrP^CWD^-positive brain homogenate to assess the sensitivity of RT-QuIC (i.e., the ability to correctly identify a true positive sample) across a range of soil textures. In the second experiment, we tested the same soil textures without adding PrP^CWD^-positive brain homogenate to evaluate the specificity of the assay (i.e., the ability to correctly identify a true negative sample).

## Materials and Methods

### Soil Textural Classification

Texture is a fundamental soil property that influences gas exchange, microbial activity, nutrient cycling, water retention, and susceptibility to erosion.^68–70^ It serves as a qualitative classification tool used to assign soils to classes based on the proportions of the silt, sand, and clay particle sizes. The U.S. Department of Agriculture (USDA) classifies soils into 12 major textural classes (Figure 1).^71^

**Figure 1:**
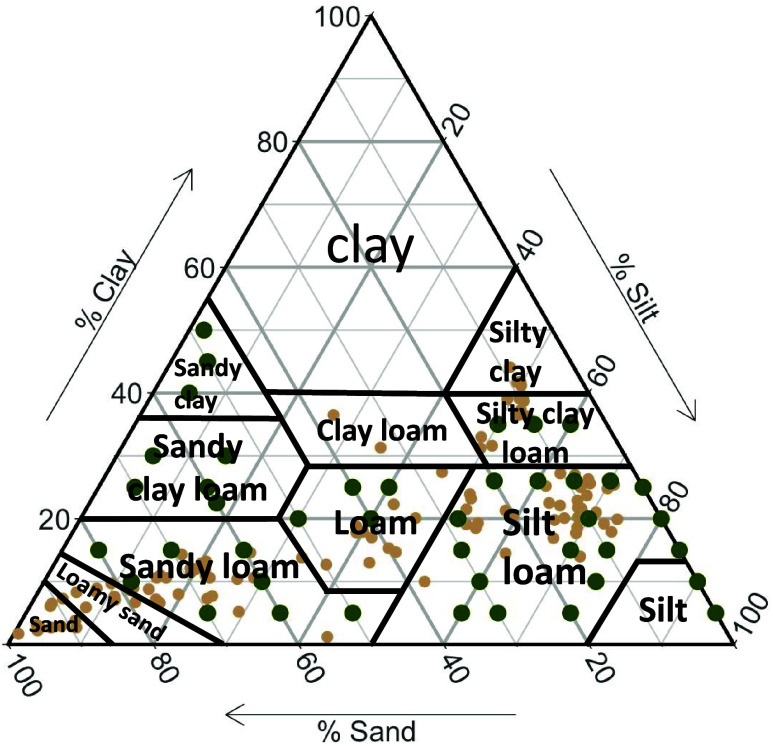
Soil textural triangle illustrating common soil textures found in Iowa County, Wisconsin, U.S.A., and soil textures generated for this study based on percent composition of sand, silt, and clay. The common soil textures are indicated by beige dots, and the 42 soil textures generated for this study are indicated by green dots.

For this study, we selected a subset of soil textures to evaluate the impact of the texture on the sensitivity and specificity of RT-QuIC. Specifically, we focused on the silt loam, sandy loam, loam, and silty clay loam textural classes that are dominant in southwestern Wisconsin, along with less common classes such as silt, sandy clay, and sandy clay loam (Figure 1).^72,73^ Although loamy sand and sandy soils are prevalent in southwestern Wisconsin, these textures were omitted because published studies and preliminary results indicate that sand-rich soils have minimal influence on prion detection and RT-QuIC performance compared to clay-dominant soils.^29,34,74,75^ The soil textures used in this study represent various ratios of clay, sand, and silt within these studied classes (Supporting Information Table S1).

Additionally, we tested several extreme soil textures, including pure silt, clay, and sand as well as silty clay loam and silty clay, which consisted only of silt and clay. These fractions were initially investigated to evaluate the effects of extreme compositions, particularly those that varied in the clay content. However, due to poor recovery outcomes, these soil textures were excluded from subsequent modeling efforts. Detailed sensitivity and specificity data for these textures are provided in Supporting Information Table S1.

### Preparation of Soils

We created the experimental soils using repository Plano soil from Arlington Farm in Arlington, Wisconsin, U.S.A. Powder X-ray diffraction (XRD) analysis was performed on three samples of the bulk Plano soil by K/T GeoServices using a Siemens D500 automated powder diffractometer (Supporting Information Table S3, Supporting Information Figures S3 and S4). Mineralogical characterization indicated that the Plano soil contained 20% sand, 54% silt, and 26% clay. The soil also tested negative for PrP^CWD^ in three rounds of PMCA (four replicates; Supporting Information Figure S1). The soil was fractionated into its silt and clay components using gravity-based particle separation,^76^ which was subsequently used to create 42 soil texture variations. Due to the mechanism of this method, sand was successfully isolated but not recovered. Instead, sand was sourced from 2 mm-0.05 mm laboratory-grade sand (Fisher Scientific, cat. no. S25–500, Waltham, MA, U.S.A.).

### Protein Misfolding Cyclic Amplification (PMCA)

To confirm that the source soil used in the experiments was PrP^CWD^-negative, the PMCA method was performed as previously described.^77–79^ The PMCA substrate was prepared using brain extracts from homozygous Tg1536 mice that were homogenized at a concentration of 10% (w/v) in PMCA conversion buffer (1X phosphate-buffered saline (PBS), 150 mM NaCl, 1% Triton X-100, and protease inhibitor cocktail (Roche, Basel, Switzerland)). The PMCA reaction consisted of 90 μL of PMCA substrate mixed with 10 μL of the Plano soil homogenate. For the first PMCA round, this mixture was subjected to 144 cycles of incubation and sonication (each PMCA cycle consisting of 29 min and 40 s of incubation, and 20 s of sonication). The resulting sample was subjected to two additional rounds of PMCA (96 cycles each) by mixing 10 μL of the PMCA product of each round with 90 μL of fresh PMCA substrate. PMCA products were treated with proteinase K (PK) as described in previous publications^77–79^ and examined by Western blot. Controls included serial dilutions of a CWD-positive brain homogenate of known PMCA activity (positive controls), and four unseeded reactions (negative controls).

### CWD-Positive Tissue Source and Preparation

A CWD-positive brain sample was collected from the obex of a hunter-harvested free-ranging white-tailed deer (*Odocoileus virginianus*) in Wisconsin (*PRNP* 96GG genotype; sample ID: 131710). Infection status was confirmed by ELISA using a USDA-approved protocol at the Wisconsin Veterinary Diagnostic Center, Madison, Wisconsin, U.S.A. The ELISA assay was conducted using a commercial Transmissible Spongiform Encephalopathy Antigen Test kit (Bio-Rad, catalogue no. 12004413, Hercules, CA, U.S.A.), following the manufacturer’s instructions. Brain tissue samples were homogenized at a concentration of 10% (w/v) in PBS (i.e., 100 mg brain in 900 μL PBS), followed by sequential enzymatic degradation. Degradation was conducted using an adapted method described by Carlson et al. with minor modifications.^80^ Specifically, in lieu of Freon-113, we used 2H,3H-perfluoropentane (Matrix Scientific, catalogue no. 003774, Columbia, SC, U.S.A.) at the same proportion relative to the aqueous phase described in the original method.

### Soil Spiking Experiments

We conducted spiking experiments to evaluate the sensitivity and specificity of RT-QuIC across 42 soil textures within silt loam, sandy loam, loam, silty clay loam, sandy clay, sandy clay loam, and silt textural classes (Supporting Table S1) using either a degraded CWD-positive brain homogenate (0.1 mg/mL, serially diluted in ultrapure water (18 MΩ H_2_O)) or an ultrapure water control. For each textural variation, 22 subsamples were prepared (11 for sensitivity and 11 for specificity). For the sensitivity experiments, each subsample (50 mg each) was spiked with 50 μL of the degraded CWD-positive brain homogenate (Supporting Figure S2). For the specificity experiments, each subsample was spiked with 50 μL of 18 MΩ H_2_O. Following the addition of spiking material, samples were vortexed for 20 s and incubated at 37 °C for 24 h as described by Saunders et al. to promote prion adsorption prior to prion extraction using the methods described below.

### Prion Extraction from Soils

After incubation, we extracted prions from each subsample by adding 400 μL of 18 MΩ H_2_O, followed by vortexing. Samples were then mixed (1400 rpm, 25 °C, 1 h) and centrifuged (8000*g*, 10 min). The resulting supernatant (300 μL) was collected, and the soil pellets were further extracted with an additional 300 μL of 18 MΩ H_2_O. Samples were vortexed, mixed (1400 rpm, 25 °C, 30 min), and centrifuged (8000*g*, 10 min). The second collection of supernatants (300 μL) was combined with the first, yielding a total volume of 600 μL per subsample. Supernatants were further clarified by centrifugation (8000*g*, 10 min), and 550 μL of the clarified supernatant was collected and transferred to a new sterile 1.5 mL microcentrifuge tube. To concentrate any PrP^CWD^ present in a sample, 500 μL of 23.1 mM sodium phosphotungstate hydrate (Na-PTA) at pH 7.1 (Sigma-Aldrich, catalogue no. 496626, St. Louis, MO, U.S.A.) was added to the supernatant, and the samples were incubated overnight at 4 °C. The following day, samples were centrifuged (16,000*g*, 4 °C, 30 min), and the resulting pellet was rinsed with a 1:1 solution of 18 MΩ H_2_O and 23.1 mM Na-PTA, followed by centrifugation (16,000*g*, 4 °C, 30 min). The final pellet was resuspended in 50 μL RT-QuIC sample buffer (0.1% sodium dodecyl sulfate (SDS) in 1X phosphate-buffered saline (PBS) with N-2 cell culture supplement (Thermo Fisher, catalogue no. 17502048, Waltham, MA, U.S.A.)) using a Qsonica Q700 cup horn ultrasonicator (Amplitude 36, 1 min). A total volume of 2 μL of each resuspended subsample extract was used to seed a reaction well of a clear-bottom 96-well plate with black sides (Thermo Fisher, catalogue no. 265301, Waltham, MA, U.S.A.) with eight technical replicates per sample. Four replicates of PrP^CWD^ positive brain were plated as positive controls, and four replicates of sample buffer as negative controls were also included on each plate.

### Real-Time Quaking-Induced Conversion (RT-QuIC) Assay

The *in vitro* prion amplification assay, RT-QuIC, was performed following the methodology described by Orrù et al. and incorporated sodium iodide as described by Metrick et al. with minor modifications. Sample extracts and controls (2 μL) were added to each corresponding well of a 96-well microplate that contained 98 μL of RT-QuIC reaction mixture (0.1 mg·mL^–1^ 90–231 recombinant hamster prion protein (produced as previously described^49^), 1.0 mM ethylenediaminetetraacetic acid, 300 mM sodium iodide, 20 mM sodium phosphate, and 10 μM Thioflavin T). The 96-well plates were then sealed using clear adhesive plate seals (Fisher Scientific, catalogue no. 08408240, Waltman, MA, U.S.A.) and inserted into microplate-compatible spectrophotometers capable of heating, shaking, and fluorescence monitoring (BMG FLUOstar, Cary, NC, U.S.A.) with the following instrument settings: 42 °C, double orbital pattern shaking at 700 rpm with 60 s shake/60 s rest cycles, fluorescent scans (λ_excitation_ = 448 nm, λ_emission_ = 482 nm) every 15 min, at a gain of 1600, and a total run time of 48 h. During each 15 min interval cycle, the assay produced a real-time readout of the relative fluorescent units (RFUs) that were generated through the recognition of Thioflavin-T binding to misfolded prion protein aggregates in each well. We then used these raw RFUs to calculate the time-to-threshold (TTT) using the MARS Data Analysis Software (BMG LABTECH, V5.03). The TTT (i.e., the time above which fluorescence amplification is determined to be distinguishable from the background fluorescence) was calculated by adding ten times the standard deviation of the RFU values from cycles 3–14 to the mean of RFU values from cycles 3–14.^62^ This method accounted for baseline variation among wells observed during the first few intervals.

### Sensitivity and Specificity Analyses

To determine the sensitivity and specificity of RT-QuIC, we derived an optimal TTT cutoff for each of the 42 soil textures using the combined data sets from the sensitivity and specificity experiments and applied this cutoff to assign binary outcomes to individual wells for each experiment. To achieve this, we fitted receiver operating characteristic (ROC) curves using the roc­() function from the pROC package^81^ (version 1.18.5) in RStudio (version 4.4.1). We applied the “closest to the top left” method to select the cutoff point on the ROC curve where sensitivity and specificity are optimally balanced.^81^ Each soil texture had its own TTT cutoff (Supporting Table S1), which was subsequently used to assign binary outcomes to the TTT data using the dplyr package^82^ (version 1.1.4). For both sensitivity and specificity experiments, we assigned a binary outcome of 1 (prion seeding activity) to wells with a TTT value that was below the cutoff. A binary outcome of 0 (no prion seeding activity) was assigned to wells with a TTT value above the cutoff or that did not reach the threshold within the 48 h assay. The resulting data set for each soil texture was updated with the binary outcomes and separated by experiment for further analysis. We then evaluated each binary outcome to classify whether a well had true or false seeding. For the sensitivity experiment, a seeding value of 1 accurately indicated the presence of prion seeding, while a seeding value of 0 indicated a false negative. For the specificity experiment, a seeding value of 1 falsely classified a negative sample as positive (false positive), while a seeding value of 0 indicated the absence of prion seeding (true negative). To evaluate the effects of % clay and % silt on the probability of true seeding (sensitivity) and false seeding (1 – specificity), we applied generalized additive models (GAMs) using the mgcv package^83^ (version 1.9–1). The application of GAMs allowed for nonlinear relationships between the predictors and the response variable, providing a rigorous method for modeling interactions between continuous environmental variables.

We assumed a Bernoulli distribution for the binary outcomes, *y*_*i*,*j*_, in each experiment (i.e., *y*_*i*,*j*_ ∼ *Bernoulli*(*p*_*i*,*j*_)) and modeled *p*_*i*,*j*_, the probability of seeding. We first created a null model that served as the baseline model with no predictors included. This model assumed that all wells had the same probability of seeding, independent of the predictors, as shown in the following equation

$$p_{i,j=g^{-1}\left(\beta_{0}\right),}$$

where *p*_*i*,*j*_ is the seeding probability in the *i*th well of the *j*th plate, g^–1^ is the inverse logit function, and β_0_ is the intercept. We used the results from this model to determine the significance of the smooth terms.

Next, we modeled the predictors as independent, additive smooth terms, represented by the following equation

$$p_{i,j}=g^{-1}\left(\beta_{0}+f_{1}\left(x_{1,i,j}\right)+f_{2}\left(x_{2,i,j}\right)\right)$$

where *f*_1_(*x*_1,*i*,*j*_) and *f*_2_(*x*_2,*i*,*j*_) are smooth terms for % silt (*x*_1_) and % clay (*x*_2_), respectively, and all other parameters and variables are as previously defined. We will refer to this model as the independent smooth model hereafter.

Additionally, we used a tensor product joint smooth function to model the interaction between % silt and % clay jointly in a flexible, nonlinear model, represented by the following equation

$$p_{i,j}=g^{-1}p_{i,j}=g^{-1}(\beta_{0}+f_{1}\left(x_{1,i,j},x_{2,i,j}\right)$$

where *f*_1_ represents the tensor product smooth function that models the interaction between % silt *(x*_1_) and % clay (*x*_2_), and all other parameters and variables are as previously defined. We will refer to this model as the joint smooth model hereafter. Overall, these two models provided a comprehensive framework that permitted us to capture both the individual and combined effects of silt and clay components on the probability of true or false seeding.

### Modeling the Optimal Time-to-Threshold (TTT) Cutoff

To evaluate the effects of % clay and % silt from each of the 42 soil textures on the optimal TTT cutoff, we applied GAMs using the mgcv package^83^ (version 1.9–1) in RStudio (version 4.4.1). To account for the positive and right-skewed nature of the TTT, we applied a γ distribution with a log link for the response variable, TTT cutoff. We applied the same three model structures described above: null, independent smooth, and joint smooth, with the response variable *t*_i_ representing the TTT cutoff for the i^th^ soil texture and g^–1^ representing the inverse log link function. Together, these models provided a comprehensive framework for evaluating both the individual and combined effects of silt and clay components on the relationship between soil texture and the optimal TTT cutoff.

### Model Selection and Prediction Surfaces

For model selection, we used the Akaike information criterion (AIC) to compare the performance of the null, independent smooth, and joint smooth models for both the sensitivity and specificity experiments, as well as models evaluating the effects of soil textures on the optimal TTT cutoff.^84^

For the sensitivity and specificity experiments, we created a prediction surface using parameter estimates from the AIC-selected top model by computing the fitted probabilities of seeding across the range of the observed silt and clay values. Additionally, we created a standard error surface to demonstrate the precision of our predictions. These surfaces were visualized using a heat map created with geom_tile­() from the ggplot2 package^85^ (version 3.5.1) to illustrate the interaction between the two variables and their effects on seeding probability.

To visualize the joint relationship between the silt and clay components from the soil textures and the optimal TTT cutoff, we created a two-dimensional prediction surface by computing the predicted TTT cutoff values on the log scale across a grid of the silt and clay values. Additionally, we created a standard error surface to demonstrate the precision of our predictions. These surfaces were visualized using the ggplot2 package^86^ (version 3.5.1), allowing for an interactive assessment of how these soil components jointly influenced the optimal TTT cutoff.

## Results

### Model Selection

Initial model selection supported the inclusion of smooth terms (Table 1). Additionally, for both the sensitivity and specificity data sets, the model selection procedure selected the joint smooth model as the most parsimonious model (Table 1). For the optimal TTT cutoff data, the AIC model selection procedure selected the joint smooth model as the most parsimonious model (Table 1).

**Table 1:** Model Selection Results for RT-QuIC Sensitivity, Specificity, and Optimal Time-to-Threshold (TTT) Analyses^a^

model	AIC	△*AIC*	relative likelihood
sensitivity models			
joint smooth	3185.19	0	1.0 × 10^0^
independent smooth	3344.02	158.83	3.24 × 10^–35^
null (β_0_)	3808.68	623.49	4.09 × 10^–136^
specificity models			
joint smooth	3384.03	0	1.0 × 10^0^
independent smooth	3468.22	84.19	5.24 × 10^–19^
null (β_0_)	3916.75	532.72	2.09 × 10^–116^
optimal TTT Models			
joint smooth	269.92	0	1.0 × 10^0^
independent smooth	274.14	4.22	0.12
null (β_0_)	300.54	30.63	2.23 × 10^–7^

a Akaike information criterion (AIC), Δ*AIC*, and relative likelihood measures were used to assess and compare the fit of the independent model, joint model, and null model for the sensitivity and specificity experiments, as well as the optimal time to threshold (TTT) cutoff data. A Δ*AIC* = 0 was considered the best-fitting model for the data.

### Modeling the Effects of Silt and Clay Using a Joint Smooth Model

#### Sensitivity

The joint smooth model revealed a significant, nonlinear effect of % silt and % clay on the predicted probability of true seeding (Supporting Table S2). The prediction surface (Figure 2A) showed that sensitivity was highest (≥95%) in soils containing less than 20% clay, regardless of silt content. In contrast, sensitivity declined (≤75%) in soils with high clay (>20%) and low silt (<60%), with the lowest predicted sensitivity values (50%–60%) observed in soils with low silt (2%–5%) and high clay (>20%). Interestingly, soils containing 25%–26% clay retained moderately high sensitivity (≥70%) when silt content exceeded 50%.

**Figure 2:**
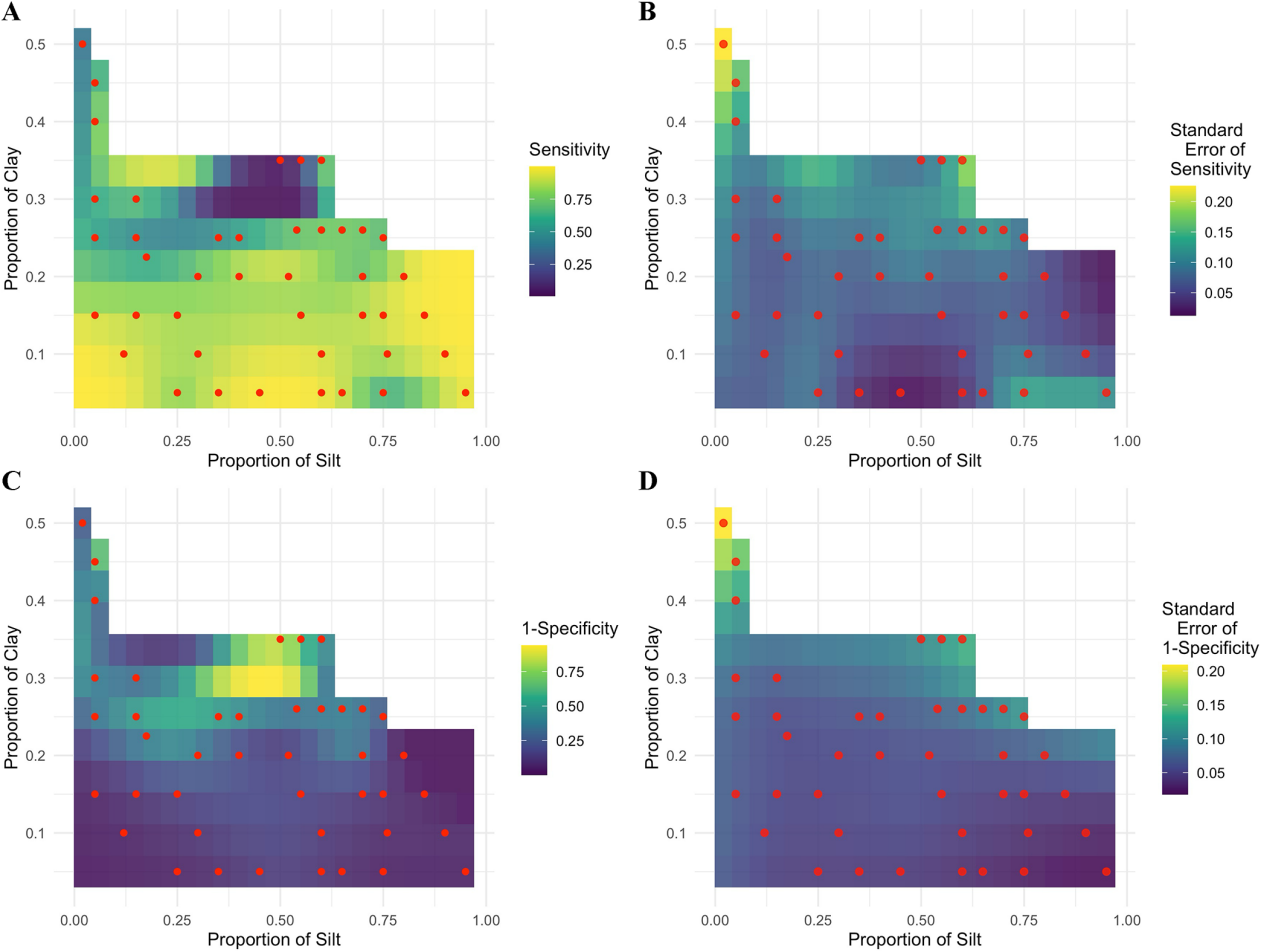
Predicted probability and standard error surfaces for sensitivity and 1-specificity as functions of % silt and % clay from the joint smooth term model, with soil textures overlaid. The prediction surfaces illustrate the predicted probabilities of true seeding rate (sensitivity; A) and false seeding rate (1 – specificity; C). Standard error surfaces illustrate the variability of the predicted sensitivity (B) and 1 – specificity (D) probability across varying levels of % silt and % clay, as estimated by the generalized additive model (GAM) with joint smooth terms. Lower standard error values indicate lower variability in the predicted values. The red dots on each surface represent the 42 soil textures evaluated in this study.

The standard error surface (Figure 2B) for sensitivity predictions ranged from 0.05 to 0.20. Soils with lower clay (<20%) and moderate to high silt (25%–65%) generally had standard errors below 0.10, indicating moderate variability in the model predictions. In contrast, soils with high clay (>20%) and low to moderate silt content showed elevated standard errors, indicating greater uncertainty in the predicted values.

#### Specificity

The joint smooth term model showed that the combined effects of silt and clay had a significant, nonlinear effect on the predicted probability of false seeding (Figure 2C; Supporting Table S2). False seeding probabilities (1 – specificity) were generally low (<30%) in soils containing less than 20% clay, regardless of silt content. Midrange probabilities (40%–50%) were observed as clay content increased to 25%, and when silt content remained below 75%. High false seeding probabilities (>50%) occurred in soils with clay content exceeding 25% and silt content below 65%.

The standard error surface (Figure 2D) ranged from approximately 0.01 to 0.20, indicating moderate to high variability in model predictions. Soils with high clay and low silt contents exhibited elevated standard errors, indicating greater uncertainty in the predicted false seeding probabilities. In contrast, soils with lower clay and silt contents showed lower standard errors, indicating a lower variation in the model results. Overall, the standard error surface suggested that predictions are less precise in clay-dominant soils and that the interaction between silt and clay impacts the estimated variability across the prediction surface.

### Modeling the Effects of Silt and Clay on the Optimal Time-to-Threshold (TTT) Cutoff Using a Joint Smooth Model

The observed variation in the TTT cutoffs (from 14 to 48 h) motivated us to model the interaction between silt and clay to assess how soil textures influenced the optimal TTT cutoff, which was selected to maximize both sensitivity and specificity for each soil texture. The joint smooth term model revealed a significant, nonlinear effect of silt and clay on the optimal TTT cutoff. Soils with high clay (>25%) and low to moderate silt content (2%–15%), or soils with low clay (<20%) and high silt content (>60%), generally had optimal TTT values of 35 h or less (Figure 3A). In contrast, the TTT cutoff values exceeded 35 h in soil containing less than 22% clay and silt ranging from 5% to 60%. These findings suggest that optimal TTTs vary across soil textures and that the cutoff maximizing the sensitivity and specificity of RT-QuIC is reduced with increasing clay.

**Figure 3:**
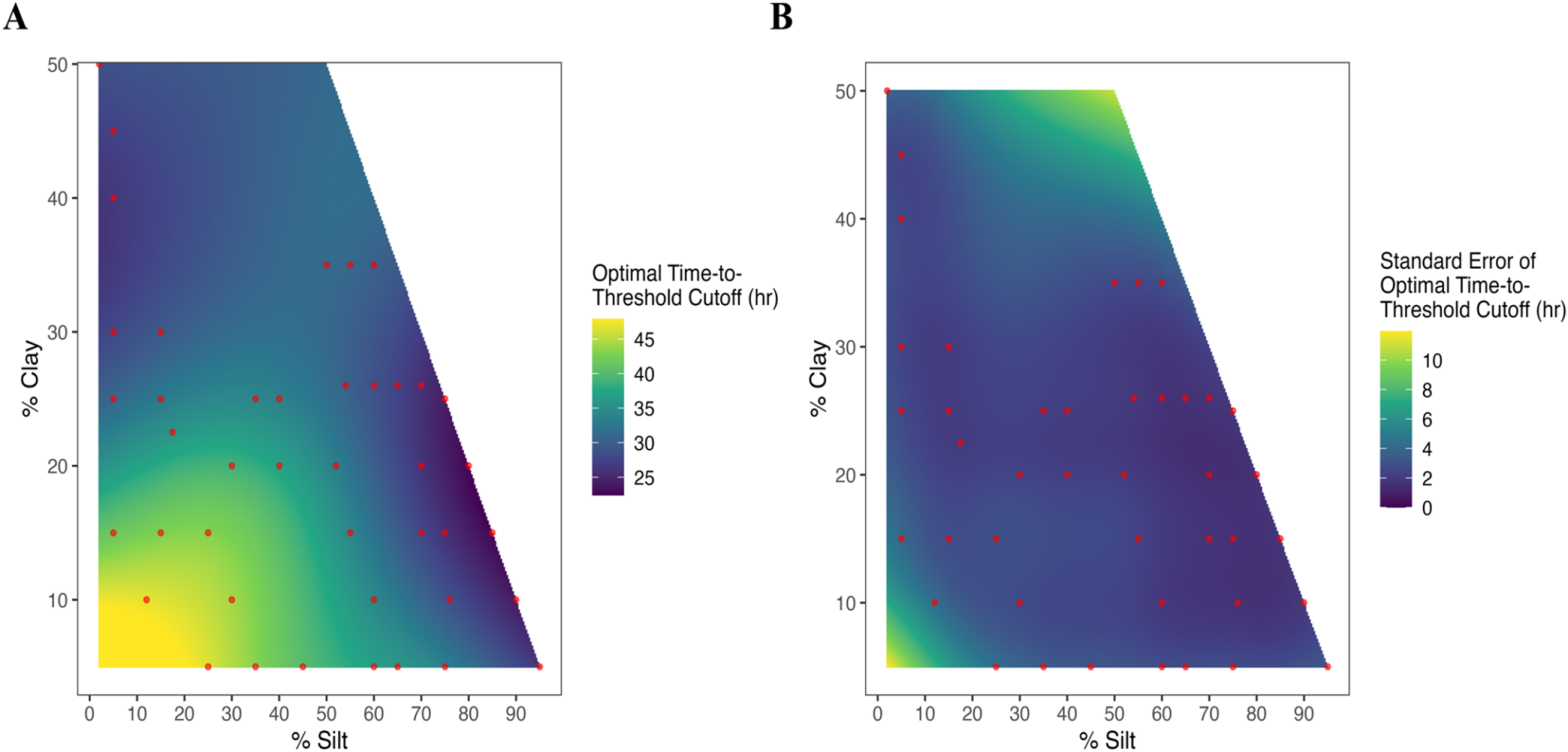
Modeled surface for the optimal time-to-threshold (TTT) cutoffs as functions of % silt and % clay from the joint smooth term model. The 2D surface plot illustrates the predicted optimal TTT cutoff values across varying levels of % silt and % clay, as estimated by the generalized additive model (GAM), with joint smooth terms (A). The standard error surface illustrates the uncertainty associated with the predicted optimal time-to-threshold (TTT) cutoff values. Lower standard error values indicate lower variability in the predictions (B). The red dots represent the 42 soil textures evaluated in this study.

The standard error surface (Figure 3B) for the optimal TTT cutoff prediction values ranged from 0 to 8 h. Overall, the predicted TTT values generally had standard errors below 4 h, indicating low uncertainty and high precision in the model predictions.

## Discussion

A key objective of this study was to evaluate the influence of soil texture on the sensitivity and specificity of prion detection using the RT-QuIC assay. Soil texture is known to play an important role in prion retention and environmental persistence,^3,29,40^ which can affect transmission risk and environmental surveillance in CWD-endemic regions.^39,40^ In this study, we found that the combined effects of silt and clay influenced PrP^CWD^ detection, particularly in soils with a higher clay content, resulting in reduced sensitivity, increased false seeding probabilities, shorter TTT cutoffs, and greater uncertainty in model predictions. Despite the increasing use of RT-QuIC for environmental PrP^CWD^ surveillance, this study is the first to systematically evaluate how variation in soil texture affects RT-QuIC performance by using a standardized extraction method. These findings suggest that soil texture may be an important yet underrecognized factor affecting the reliability of environmental prion detection.

Soils with a high clay content may reduce RT-QuIC sensitivity by promoting strong adsorption of prions to clay mineral surfaces. This binding occurs through electrostatic and hydrophobic interactions, as clay-rich soils often exhibit high cation exchange capacity, complex mineral surfaces, and an abundance of polar binding sites.^34,40,87^ These interactions may limit prion desorption during simple extraction methods, thereby reducing the availability of prions to act as seeds for subsequent template-mediated misfolding in the RT-QuIC reaction. Interestingly, soils with a higher clay content were also associated with increased false positives, possibly due to effects on the intrinsic misfolding behavior of the recombinant PrP substrate used in the RT-QuIC assay. Nevertheless, the exact mechanisms underlying this effect remain unclear and could be determined with an additional investigation.

In this study, we also found that soil texture composition, particularly the relative proportions of silt and clay, had a strong, nonlinear effect on RT-QuIC sensitivity and specificity. Soils with lower clay content (<20%) were associated with a higher probability of true seeding and a lower probability of false seeding, whereas soils with higher clay and low to moderate silt content exhibited the opposite trend. Overall, these findings suggest that soil textures with low clay and moderate to high silt contents have a limited impact on RT-QuIC performance. In contrast, clay-dominant soils can substantially reduce sensitivity and specificity, making reliable PrP^CWD^ detection more challenging, particularly when a single extraction approach is used.

Silt and clay also had a strong, nonlinear influence on the optimal TTT cutoff used to classify RT-QuIC reactions as positive or negative. Soils with low clay and moderate to high silt contents generally allowed for longer TTT cutoffs, whereas soils with higher clay contents required shorter TTT cutoffs. These findings suggest that clay-rich soils may require more conservative TTT cutoffs to maintain an optimal balance between sensitivity and specificity, particularly to reduce the false seeding rates.

In addition to the soil textures included in the predictive models, we evaluated extreme soil compositions outside the modeling range, including pure silt, sand, and clay as well as combinations of only silt and clay. These soils produced highly variable RT-QuIC responses, which prevented reliable model predictions (Supporting Information Table S1). To address these issues, highly restricted TTT cutoffs were applied, introducing uncertainty in the interpretation of the sensitivity and specificity data. As a result, these textures were excluded from final models. This variability may reflect strong prion adsorption, heterogeneous extraction efficiency, or other matrix-associated effects that influence RT-QuIC amplification. Nevertheless, their variable assay behavior further supports the conclusion that soil texture may substantially influence RT-QuIC performance.

These findings suggest that while RT-QuIC detection of PrP^CWD^ is effective in certain soil textures, the soil composition can influence the optimal TTT cutoff required to balance sensitivity and specificity. Notably, uncertainty in assay performance increases in clay-rich soils, highlighting the importance of conducting soil characterization prior to analysis of field samples in environmental prion surveillance efforts. Such characterization could help researchers anticipate how site-specific properties may influence assay performance and interpret results more reliably, particularly in clay-rich or compositionally heterogeneous environments.

Overall, this study underscores the importance of accounting for soil variability when interpreting results from RT-QuIC and potentially other prion detection assays. Refining extraction protocols to address inhibitory effects and improve desorption techniques may improve the detection reliability across diverse soil types. Additionally, we provided texture-specific TTT cutoffs that may serve as reference values for future studies seeking to optimize the RT-QuIC classification thresholds. Although further validation is warranted, this approach offers a framework for improving the consistency and interpretability of the RT-QuIC results in environmental samples.

This study used a simplified and systematic experimental design to isolate the texture-associated effects on prion recovery and RT-QuIC detection, relying on a single soil source and a single water-based extraction method. Although water-based extraction approaches have been used in prior studies, they have known limitations in clay-rich soils.^75^ Clay minerals can retain prions through interactions with the N-terminal region, electrostatic interactions with protein side chains, and ionizable groups along the C-terminal region,^74^ reducing recovery efficiency and assay sensitivity.

Using a single parent soil to generate a range of soil textures with different levels of clay content resulted in a fixed mineralogy across all of the samples. While this allowed for controlled comparisons, it does not fully capture the natural diversity of clay mineral types that may influence the prion retention and extraction. Furthermore, alternative extraction methods (i.e., detergent-based approaches) may improve desorption in certain soil textures and potentially alter RT-QuIC performance.^75^ For these reasons, the findings presented here should be considered preliminary, and future work incorporating diverse soil mineralogies and multiple extraction methods could be beneficial. Sand was excluded from the modeling analysis because of its minimal predicted influence on assay sensitivity and specificity, enabling a more targeted examination of the effects of silt and clay.

Future research aimed at improving our understanding of how soils influence PrP^CWD^ detection, false positives, and environmental transmission could expand upon this work by evaluating a broader range of extraction methods and natural soil textures, and examining additional soil properties, such as OM or pH, that may influence assay performance. Validation studies may also help determine how RT-QuIC detection of PrP^CWD^ seeding activity across different soil textures relates to environmental infectivity and transmission potential through bioassay approaches. As CWD continues to expand geographically, the demand for robust environmental surveillance tools has grown. This study provides an initial framework for evaluating the reliability of RT-QuIC for PrP^CWD^ detection in complex soil matrices and supports its potential utility in soil-based prion surveillance efforts.

## Supplementary Material



## Data Availability

Data supporting the findings of this study^88^ are publicly available via USGS ScienceBase: 10.5066/P13AJ49L.

## Notes

Experimentally formulated soil textures (Table S1); summary of smooth terms in generalized additive model (Table S2); X-ray diffraction data (weight percent) of plano soil (SL2) (Table S3); Western blot evaluating prion seeding activity in plano soil using the protein misfolding cyclic amplification (PMCA) method (Figure S1); serial dilution of a CWD-positive degraded brain homogenate used for spiking experiments (Figure S2); X-ray diffraction trace on the bulk (whole-rock) plano soil (Figure S3); and X-ray diffraction trace on the ethylene glycol (EG) solvated clay fraction (<4 μm) and air-dried (AD) clay fraction (<4 μm) of plano soil (Figure S4) (PDF)

## Abbreviations

CWD: chronic wasting disease
PrP: prion protein
PrP^CWD^: chronic wasting disease prion protein
RT-QuIC: real-time quaking-induced conversion
PMCA: protein misfolding cyclic amplification
TTT: time to threshold
RFU: relative fluorescent unit
GAM: generalized additive models
AIC: akaike information criterion
