# Supplementary material for: Digging into Soil: Effects of Soil Texture on RT-QuIC Performance for Environmental Prion Surveillance
Source: Environ Sci Technol. 2026 Jul 16;60(29):20598–608. doi: 10.1021/acs.est.6c03979 (PMC13421972; doi:10.1021/acs.est.6c03979)

# Supporting Information

# Digging into soil: effects of soil texture on RT-QuIC performance for environmental prion surveillance

*Stephanie J. Katircioglu*[1], Heather N. Inzalaco[1], Allen Herbst[2], Daniel J. Storm[3], Stuart S. Lichtenberg[4,5], Rodrigo Morales[6,7], Reece McGinn[6], Daniel P. Walsh[8] and Wendy C. Turner[9]*

[1]Wisconsin Cooperative Wildlife Research Unit, Department of Forest and Wildlife Ecology, University of Wisconsin-Madison, Madison, Wisconsin 53706, United States

[2]U.S. Geological Survey, National Wildlife Health Research Center, Madison, Wisconsin 53711, United States

[3]Wisconsin Department of Natural Resources, Eau Claire, Wisconsin 54701, United States

[4]Department of Veterinary and Biomedical Sciences, University of Minnesota, St. Paul, Minnesota 55108, United States

[5]Minnesota Center for Prion Research and Outreach, University of Minnesota, St. Paul, Minnesota 55108, United States

[6]Department of Neurology, The University of Texas Health Science Center at Houston, Houston, Texas 77030, United States

[7]Centro Integrativo de Biologia y Quimica Aplicada (CIBQA), Universidad Bernardo O'Higgins, Santiago 8370993, Chile

[8]U.S. Geological Survey, Montana Cooperative Wildlife Research Unit, Wildlife Biology Program, University of Montana, Missoula, Montana 59812, United States

[9]U.S. Geological Survey, Wisconsin Cooperative Wildlife Research Unit, Department of Forest and Wildlife Ecology, University of Wisconsin-Madison, Madison, Wisconsin 53706, United States

*Corresponding Author: Stephaniekatircioglu@gmail.com

Summary: 7 pages, 3 tables, 4 figures

**Table S1:** Experimentally formulated soil textures. This table presents each soil textural class and the different percentages of clay, silt, and sand that make up these classes. Optimal time-to-threshold cutoffs that were used to classify a binary outcome for the data from each soil texture generated using receiver operating curves (ROC) is also shown along with the new sensitivity and specificity rates after assigning the binary outcome.

| Textural class | Clay (%) | Sand (%) | Silt (%) | Optimal time-to-threshold cutoff (hours) | Sensitivity (%) | Specificity (%) |
|---|---|---|---|---|---|---|
| Silty Clay Loam | | | | | | |
| | 35 | 15 | 50 | 28.3 | 76.1 | 71.6 |
| | 35 | 10 | 55 | 34.1 | 84.1 | 75 |
| | 35 | 5 | 60 | 36.0 | 85.2 | 58 |
| | 35 | 0 | 65 | 29.5 | 93.2 | 93.2 |
| | 30 | 0 | 70 | 27.3 | 71.6 | 80.7 |
| Loam | | | | | | |
| | 25 | 40 | 35 | 34.0 | 71.6 | 78.4 |
| | 25 | 35 | 40 | 30.8 | 79.5 | 70.5 |
| | 20 | 50 | 30 | 39.7 | 93.2 | 78.4 |
| | 20 | 40 | 40 | 47.9 | 54.5 | 61.4 |
| Silt Loam | | | | | | |
| | 26 | 20 | 54 | 23.8 | 45.5 | 37.5 |
| | 26 | 14 | 60 | 32.3 | 70.5 | 70.5 |
| | 26 | 9 | 65 | 27.7 | 77.3 | 62.5 |
| | 26 | 4 | 70 | 27.2 | 89.8 | 76.1 |
| | 25 | 0 | 75 | 21.8 | 59.1 | 71.6 |
| | 20 | 28 | 52 | 28.4 | 93.2 | 81.8 |
| | 20 | 10 | 70 | 29.0 | 80.7 | 76.1 |
| | 20 | 0 | 80 | 14.3 | 97.7 | 100 |
| | 15 | 30 | 55 | 38.8 | 93.2 | 88.6 |
| | 15 | 15 | 70 | 29.3 | 83 | 85.2 |
| | 15 | 10 | 75 | 31.1 | 96.6 | 86.4 |
| | 15 | 0 | 85 | 20.2 | 95.5 | 93.2 |
| | 10 | 30 | 60 | 28.9 | 95.5 | 92 |
| | 10 | 14 | 76 | 26.8 | 81.8 | 90.9 |

| | | | | | | |
|---|---|---|---|---|---|---|
| | 5 | 35 | 60 | 33.2 | 97.7 | 94.3 |
| | 5 | 30 | 65 | 28.1 | 94.3 | 89.8 |
| | 5 | 20 | 75 | 47.8 | 64.8 | 93.2 |
| Sandy Clay Loam | | | | | | |
| | 30 | 65 | 5 | 28.5 | 73.9 | 71.6 |
| | 30 | 55 | 15 | 30.9 | 68.2 | 68.2 |
| | 25 | 70 | 5 | 25.8 | 61.4 | 76.1 |
| | 25 | 60 | 15 | 31.9 | 25 | 26.1 |
| | 22.5 | 60 | 17.5 | 34.5 | 47.7 | 44.3 |
| Sandy Clay | | | | | | |
| | 50 | 48 | 2 | 29.5 | 38.6 | 73.9 |
| | 45 | 50 | 5 | 29.0 | 62.5 | 50 |
| | 40 | 55 | 5 | 23.57 | 61.4 | 58 |
| Sandy Loam | | | | | | |
| | 15 | 80 | 5 | 43.1 | 94.3 | 94.3 |
| | 15 | 70 | 15 | 47.9 | 88.6 | 89.8 |
| | 15 | 60 | 25 | 46.7 | 85.2 | 85.2 |
| | 10 | 78 | 12 | 42.2 | 88.6 | 89.8 |
| | 10 | 60 | 30 | 44.9 | 92 | 93.2 |
| | 5 | 70 | 25 | 47.8 | 87.5 | 96.6 |
| | 5 | 60 | 35 | 37.2 | 87.5 | 89.8 |
| | 5 | 50 | 45 | 46.6 | 95.5 | 81.8 |
| Silt | | | | | | |
| | 10 | 0 | 90 | 26.5 | 100 | 97.7 |
| | 5 | 0 | 95 | 23.6 | 96.6 | 94.3 |
| | 0 | 0 | 100 | 29.9 | 96.6 | 97.7 |
| Silty Clay | | | | | | |
| | 50 | 0 | 50 | 47.0 | 0 | 23.9 |
| | 40 | 0 | 60 | 34.2 | 71.6 | 46.6 |
| Clay | | | | | | |
| | 100 | 0 | 0 | 48 | 0 | 100 |
| | 95 | 0 | 5 | 43.2 | 94.3 | 100 |
| | 85 | 0 | 15 | 39.2 | 100 | 100 |
| Sand | | | | | | |
| | 0 | 100 | 0 | 46.8 | 69.3 | 85.2 |

**Table S2:** Summary of smooth terms in Generalized Additive Model (GAM) for both the sensitivity and specificity experiments, as well as the optimal time-to-threshold (TTT) cutoff model. Effective degrees of freedom, reference degrees of freedom, Chi-Square ($X^2$) and the p-value are shown for the independent smooth model and the joint smooth model with the tensor product function.

| Smooth Term | Model | Test | edf | Ref. df | Chi. sq | *p*-value |
|---|---|---|---|---|---|---|
| te(X._Silt,X._Clay) | Joint smooth | Sensitivity | 22.77 | 23.04 | 388.9 | <2e-16*** |
| te(X._Silt,X._Clay) | Joint smooth | Specificity | 22.83 | 23.1 | 371.4 | <2e-16*** |
| te(X._Silt,X._Clay) | Joint smooth | Optimal TTT | 8.47 | 10.6 | n.a. | 2.35e-05*** |

**Table S3:** X-ray Diffraction Data (Weight percent) of Plano Soil (SL2).

| XRD# | Sample ID | Quartz | K-Feldspar | Plagioclase | Calcite | Pyrite | Hematite | R0 M-L I/S (90%S)* | Illite&Mica | Kaolinite | Chlorite | TOTAL |
|---|---|---|---|---|---|---|---|---|---|---|---|---|
| SL1 | LUFA 2.2 | 83.6 | 12.1 | 1.6 | 0 | 0 | 0 | 0 | 2.3 | 0.2 | 0.2 | 100 |
| SL2 | Plano | 55 | 9.4 | 15.5 | 0.4 | 0 | 0.7 | 13.1 | 4.4 | 0.9 | 0.6 | 100 |
| SL3 | Compost Base | 79.5 | 9.3 | 7.7 | 0.3 | 0.3 | 0 | 0 | 2.2 | 0.4 | 0.3 | 100 |

*R0 M-L I/S (90%S) - R0 Ordered Mixed-Layer Illite/Smectite with 90% Smectite Layers

**Figure S1:** Evaluation of prion seeding activity in Plano soil (sample #2) using the protein misfolding cyclic amplification (PMCA) method. Plano soil was evaluated for its prion content using the PMCA method as described in[78]. (A) represents PMCA analysis of serially diluted chronic wasting disease (CWD) positive brain homogenate extracts used as a positive control. (B) represents the results of unseeded reactions used as negative controls (N.C.1-N.C.4) and Plano soil (#2) that was analyzed in duplicate. This corresponds to a third PMCA round. All samples were treated with PK. The line at the right of each picture represents the molecular weight markers.

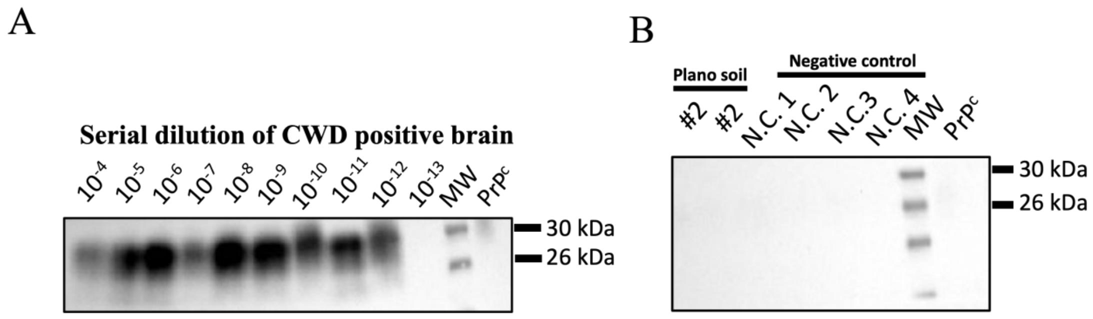

**Figure S2:** Serial dilution of CWD-positive degraded brain homogenate (BH) used for soil spiking experiments.

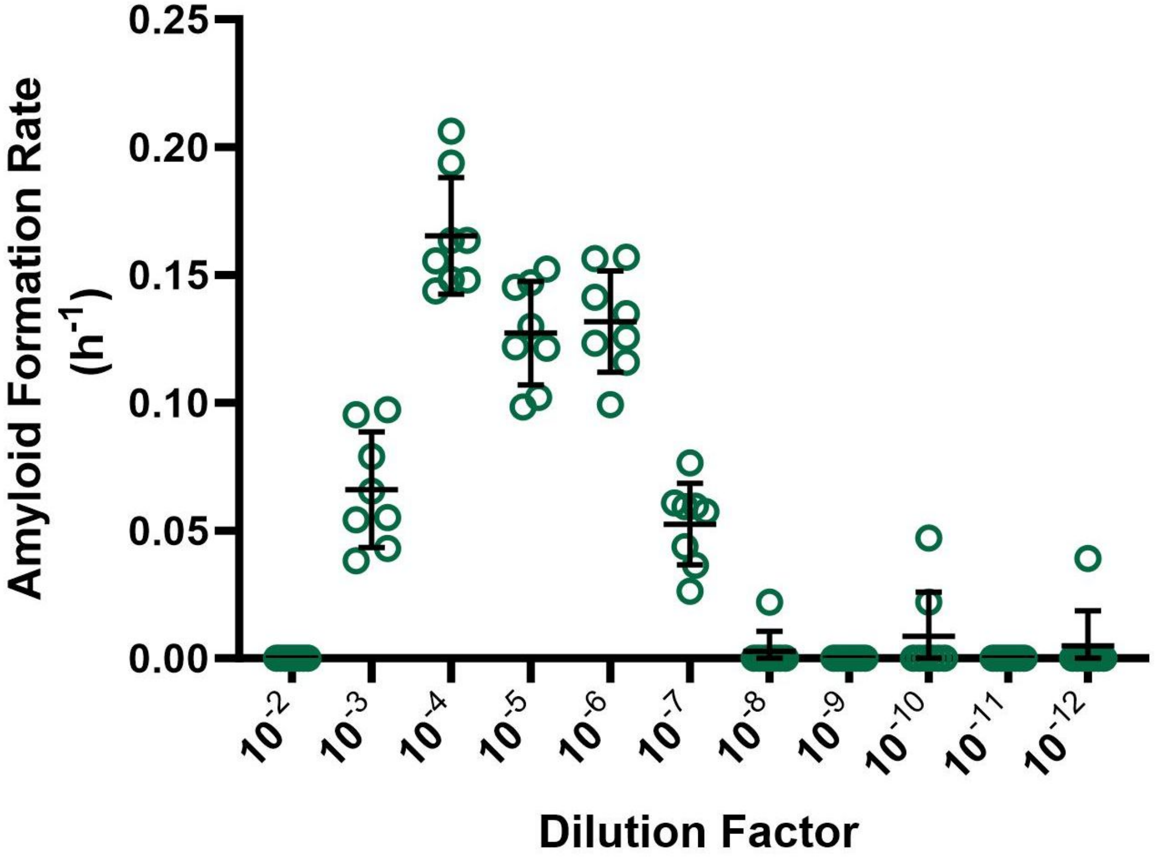

**Figure S3:** X-ray Diffraction Trace on the Bulk (Whole-Rock) Plano Soil.

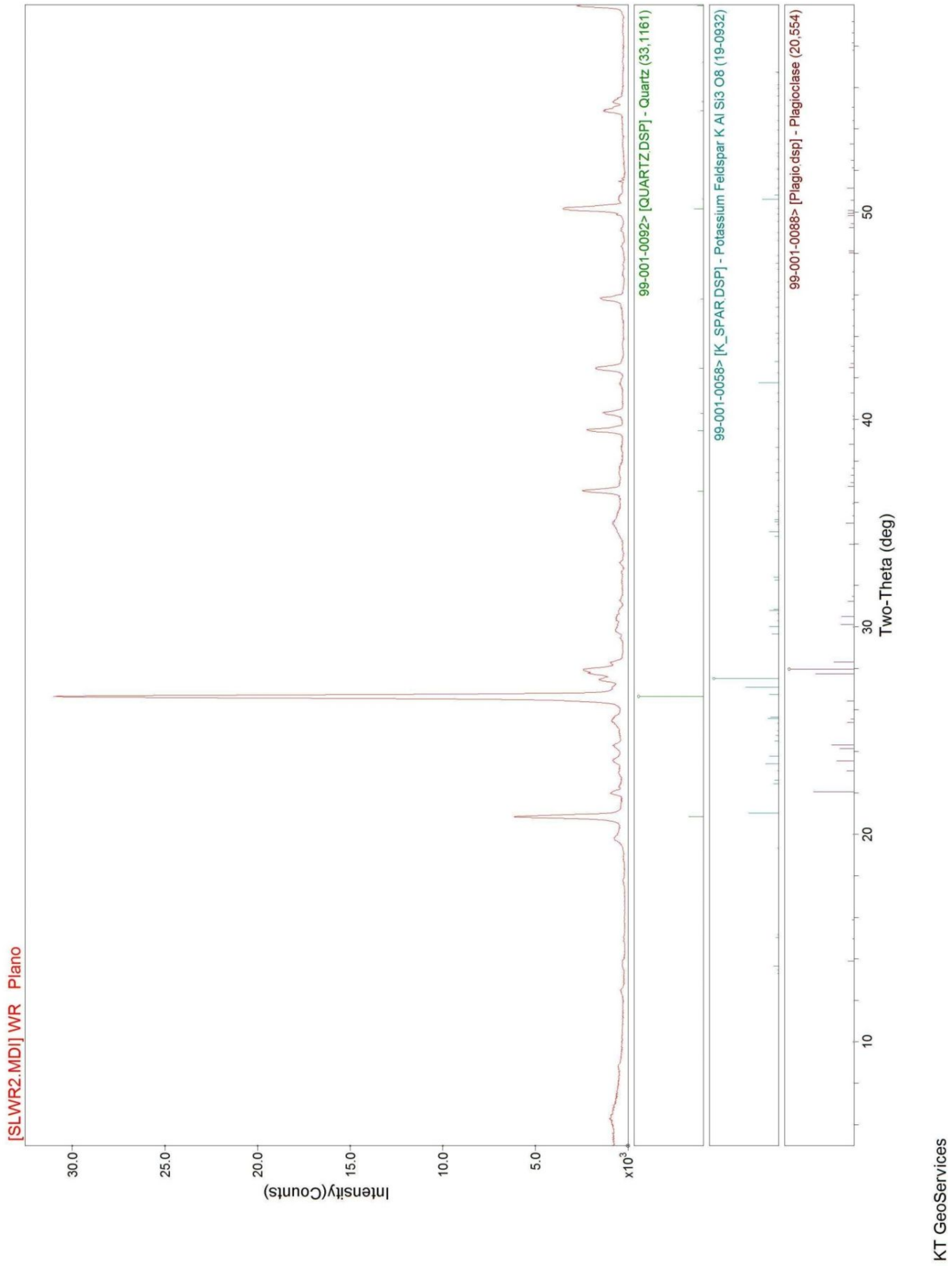

**Figure S4:** X-ray Diffraction Trace on the Ethylene Glycol (EG) Solvated Clay Fraction (<4 micron) and Air-Dried (AD) Clay Fraction (<4 micron) of Plano Soil.

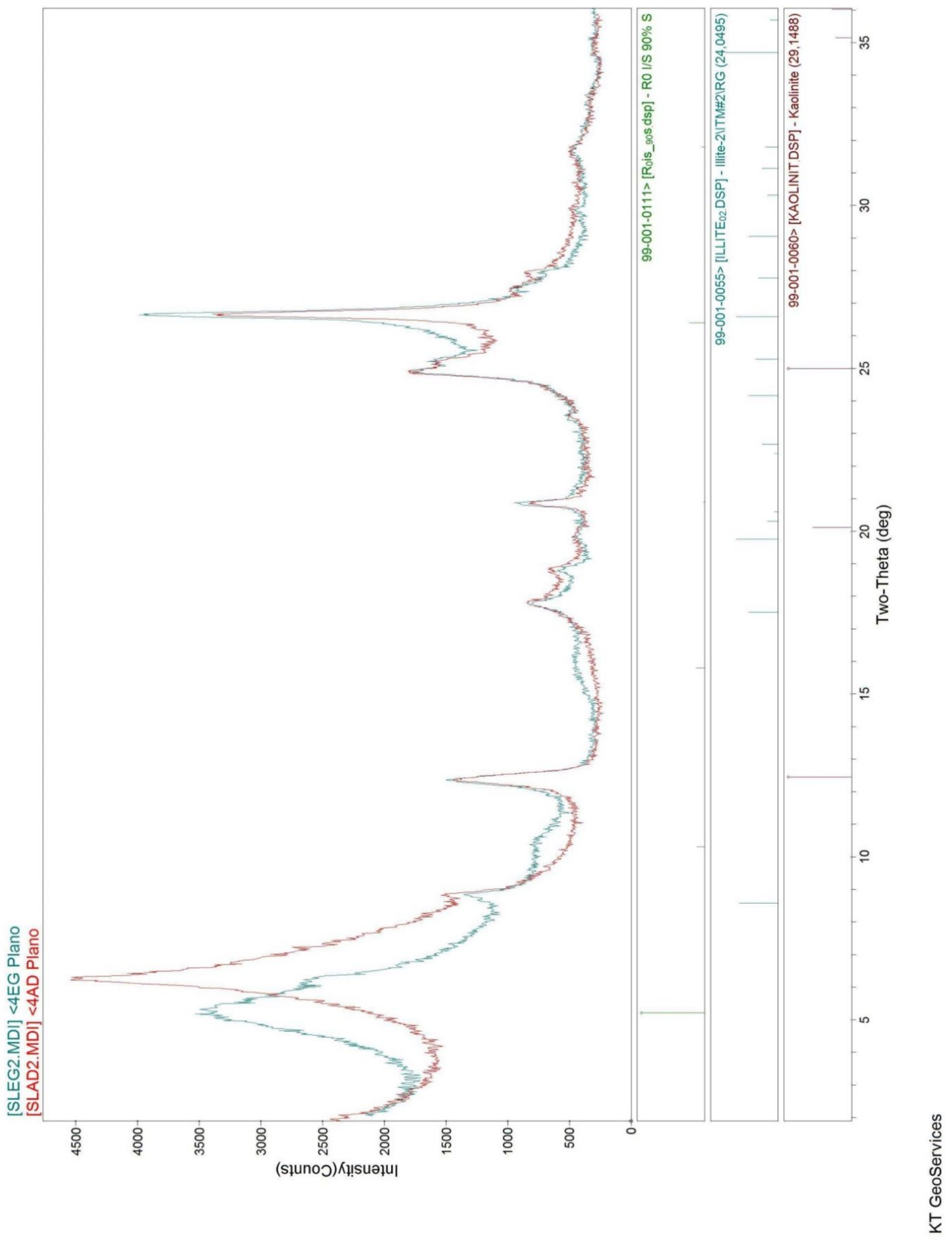

Supplement: Supplementary file 1 [file es6c03979_si_001.pdf]
